# Experimental modeling and optimization for the reduction of hexavalent chromium in aqueous solutions using ascorbic acid

**DOI:** 10.1038/s41598-021-92535-y

**Published:** 2021-06-23

**Authors:** Qammer Zaib, Hung Suck Park, Daeseung Kyung

**Affiliations:** Department of Civil and Environmental Engineering, University of Ulsan, Daehak-ro 93, Nam-gu, Ulsan, 44610 Republic of Korea

**Keywords:** Environmental sciences, Pollution remediation

## Abstract

In this study, we investigated the reduction of toxic Cr(VI) to less toxic Cr(III) using ascorbic acid in various aqueous solutions: deionized water, synthetic soft water, synthetic hard water, and real tap water. The experiments were performed using a statistical experimental design. Response surface methodology (RSM) was used to correlate Cr(VI) reduction (response variable) with experimental parameters such as initial Cr(VI) concentration, humic acid concentration, and ascorbic acid dosage. The empirical model obtained from the experiments was used to estimate and optimize the quantity of ascorbic acid required for the reduction of ≥ 99% Cr(VI) in water. The optimized dosages of ascorbic acid were predicted and experimentally validated for > 99.5% reduction of Cr(VI) (1, 10, 20, and 100 mg/L) in the solutions. Even a solution containing an initial Cr(VI) concentration of 100 mg/L was reduced in concentration ≥ 99.9% with optimal dosage of ascorbic acid (500 mg/L) in the presence of 20 mg/L humic acid. Moreover, the reaction kinetics (k_obs_-Cr(VI) = 0.71 mM^−1^ s^−1^) were sufficient to reduce the ≥ 99.9% Cr(VI) in 20 min. This study sheds new light on the effect of ascorbic acid on Cr(VI) reduction, and provides knowledge fundamental to optimize treatment of Cr(VI) contaminated water to environmentally acceptable endpoints.

## Introduction

Chromium is a commercially important metal^1^. It is frequently used in various industries such as electroplating, metal cleaning, alloying, fertilizers, paints and pigments, timber preservation, and leather tanning, among others^2–4^. This extensive usage of chromium has led to its widespread occurrence in groundwater and surface water chiefly via industrial effluent discharge^5–7^. The industrial effluents usually contain hexavalent [Cr(VI)] and/or trivalent [Cr(III)] forms of chromium, the two major oxidation states of the metal that are stable in the environment^2^. Cr(III) is an innocuous and rather essential micronutrient for living organisms and it plays a significant role in the metabolism of carbohydrates and lipids^8^. On the other hand, Cr(VI) is considerably mobile in aquatic systems and readily contaminates water bodies due to its toxic, mutagenic, and carcinogenic nature^9^. Therefore, it is essential to remove or reduce Cr(VI) in aquatic systems.

Several physicochemical treatments have been adopted to treat Cr(VI) such as ion exchange, extraction, evaporation, concentration, reverse osmosis, membrane separation, electrochemical precipitation, bio-separation, and chemical reduction^1,4,10^. Recently, adsorption and biosorption have also attracted special attention for Cr(VI) removal^11–15^. Additionally, chemical reduction is an effective option to treat chromium, due to its ability to transform highly soluble and toxic Cr(VI) to relatively less soluble and harmless Cr(III)^2,16–19^. The chemical reduction can be carried out using strong reducing agents such as hydrogen peroxide (H_2_O_2_), hydrogen sulfide (H_2_S), ferrous [Fe(II)] solution, glycerol, and/or sulfur dioxide (SO_2_)^17,20,21^. However, the usage of these chemicals is either ineffective in neutral/alkaline pH or else generates additional environmental problems with the creation of hazardous by-products^17,20^.

**Figure 1:**
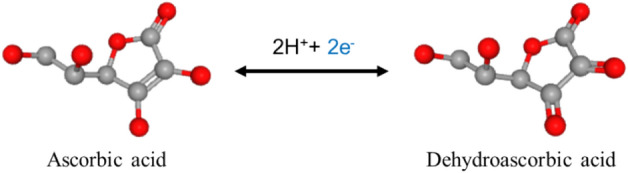
The oxidation of ascorbic acid to dehydroascorbic acid (adapted with modifications from PubChem database^22^).

Ascorbic acid (Vitamin C) is an essential micronutrient which is ubiquitous in higher animals including humans^17,23^. It is a strong biological reducing agent providing electrons when it oxidizes to dehydroascorbic acid as shown in Fig. 1^23^. Therefore, it is capable of reducing toxic metals in living organisms^24,25^. Despite the significance of ascorbic acid as a biological reducing agent, it is seldom studied for the reduction of Cr(VI) in aqueous solutions. A handful of studies have reported the effects of physicochemical parameters on the reduction of Cr(VI) using ascorbic acid in aqueous solutions^17,26^. However, these studies only examined the effect of one factor at a time, and therefore failed to demonstrate the interactive effects of the parameters on Cr(VI) reduction in aqueous systems. This shortcoming in the scientific literature could be addressed by employing Response Surface Methodology (RSM). RSM is a combination of statistical and mathematical techniques that can be helpful in evaluating the combined effects of several factors at a time on a response variable^27,28^. By applying RSM, an effective understanding of the correlation of several experimental factors and their influence on Cr(VI) reduction under limited experimental conditions is probable^27^.

Therefore, using RSM, this study aimed to investigate both the individual and combined effects of factors influencing on Cr(VI) reduction and to predict the optimal dosages of ascorbic acid required for diverse aqueous solutions for the first time. The RSM-model-predicted optimal dosages were statistically verified by analysis of variance (ANOVA) and experimentally validated by applying pre-determined amounts of ascorbic acid dosages to Cr(VI) contaminated water solutions in the presence of humic acid. The results obtained from this study could be applied to effectively treat Cr(VI) contaminated water using ascorbic acid.

## Materials and methods

### Materials

Reagent grade potassium dichromate, magnesium chloride hexahydrate, calcium chloride hexahydrate, calcium nitrate tetrahydrate, calcium carbonate, sodium sulfate, potassium bicarbonate, sodium bicarbonate, potassium dihydrogen phosphate, magnesium sulfate heptahydrate, sodium chloride, potassium chloride, and sodium nitrate were acquired from Daejung Chemicals & Metals Co., Ltd. (Korea). L-Ascorbic acid (≥ 99.5%) was purchased from Samchun Pure Chemical Co., Ltd. (Korea). 1,5-diphenylcarbohydrazide (DPC) and sulfuric acid were obtained from Kanto Chemical Co., Inc. (Japan). Acetone was provided by OIC Co., Ltd. (Korea). 50 mL DPC reagent was prepared by dissolving 250 mg of DPC in 50 mL of acetone. Unless otherwise specified, freshly prepared deionized water (DIW) with an average resistivity of 18.2 MΩ-cm was used in all experiments. Synthetic soft water (SW) and hard water (HW) were prepared according to the protocols published elsewhere^29^. Actual tap water (TW) was acquired from the drinking water supply of the laboratory where all experiments were performed.

### Batch experimentation and analysis

A stock solution of 10,000 mg/L Cr(VI) solution was prepared by dissolving the calculated amount of potassium dichromate in the deionized water. The stock solution was diluted to obtain the standard solutions and the working solutions of desired Cr(VI) concentrations for method development and reduction experiments, respectively. The reduction of Cr(VI) was carried out in 40 mL amber vials by spiking ascorbic acid to Cr(VI) in various aqueous backgrounds (i.e., DIW, presence/absence of humic acid, SW, HW, and TW). The vials were vigorously shaken for 30 min before determining the residual concentration of Cr(VI) after reduction. Several blanks and control experiments were regularly run, along with the samples, throughout the course of experimentation. All the experiments were performed in duplicate at a minimum.

The Cr(VI) concentration was determined using a UV–Vis spectrophotometer and/or colorimeter following EPA Method 7196^30^. The method quantification limit was assessed and a nearly linear correlation (R^2^: 0.999) between visible light absorbance at λ = 540 nm and Cr(VI) concentration (from 0.002 to 1 mg/L) was observed. Figures S1 and S2 (Supplementary Information) represent the standard calibration curves of total chromium and Cr(VI), respectively. The quantities of reduced chromium, i.e. Cr(III), were determined from total chromium using ICP-OES (Optima 8300, PerkinElmer, USA) as shown in Fig. S3. Please refer to ‘Quantitative verification of Cr(VI) and Cr(III) in aqueous solutions’ in Supplementary Information for details.

### Experimental design

The effects of initial Cr(VI) concentration, humic acid concentration, and ascorbic acid dosage on Cr(VI) reduction were experimentally investigated using central composite rotatable design (CCRD) and RSM, which helps construct an appropriate mathematical model for the desired response^31^. RSM comprises statistical and mathematical methods to fit models and analyze independent parameters with respect to dependent response^32^. The CCRD, based on RSM, helps assess the impacts of factors and their corresponding interactions on the response, i.e., Cr(VI) reduction in our case. Equation (1) can be used to calculate the number of experiments required for a central composite design of experiments^28^:

1 $$N = 2^{k} + 2k + n_{c}$$

where N represents the number of experiments required, k is the number of factors, and n_c_ indicates the number of experiments at the central point. The three terms in Eq. (1) represent the experimental runs at factorial points (2^k^), axial points (2k), and a central point (n_c_). The number of experiments at a central point usually varies from 2 to 6, depending upon the robustness of the design, as central points are responsible for the reproducibility of the data and experimental error^27^. From Eq. (1), three-factor experimental combinations can be examined by performing six experiments at axial points, eight at factorial points, and another six at the central point to maximize the reliability of the model. In this study, a CCRD was used to investigate the effects of ascorbic acid dosage, humic acid, and initial Cr (VI) concentration on the reduction of Cr (VI). These three factors, respectively labeled A, B, and C were studied at five levels (− α, − 1, 0, + 1, + α) as shown in Table 1. The axial, factorial, and central experimental points correspond to (− α, + α), (− 1, + 1), and (0) levels, respectively, in Table 1. Design-expert software was used to process the experimental data. The effect of factors on the response (% Cr(VI) reduction) was modeled using a fourth-order polynomial equation. The regression coefficients were evaluated using analysis of variance (ANOVA). The adequacy of the model was assessed by a determination coefficient (R^2^), adjusted determination coefficient (adj-R^2^), adequate precision, and lack-of-fit tests^31,33–35^. The model was used to assess individual and combined effects of factors on % reduction of Cr(VI) in water. It was also utilized to optimize ascorbic acid dosages for optimal reduction of Cr(VI) in various aqueous solutions such as DIW, SW, HW, and TW.

**Table 1:** Experimental factors and their levels for the reduction of Cr(VI) using ascorbic acid.

	Factors	Units	Levels				
			− α	− 1	0	1	+ α
A	Ascorbic acid	mg/L	0	101	250	399	250
B	Humic acid	mg/L	0	20	50	80	100
C	Cr(VI)	mg/L	0.1	20	50	80	100

## Results and discussion

### Model development and analysis

The experimental factors and their levels were predetermined to investigate the effects of ascorbic acid, humic acid, and initial Cr(VI) concentration (mg/L) on the reduction (%) of Cr(VI) (Table 1).The CCRD was chosen for this study because it allows extrapolation along with navigation inside the experimental design space^27^. The complete experimental design matrix is shown in Table 2. It lists twenty experimental runs comprising of various combinations of factors and their experimentally obtained responses in the aqueous solutions, i.e., Cr(VI) residual concentrations (mg/L) and Cr(VI) reduction (%). The experimental data were used to develop a quadratic empirical model to correlate the factors and one of the responses (Cr(VI) reduction (%)). The RSM model, hence obtained, after elimination of the non-significant model terms through backward elimination, is presented by Eq. (2).

2 $${\text{Cr}}\left( {{\text{VI}}} \right)\;{\text{reduction}}~\;\left( \% \right)=99.79~ + 29.71~{\text{A}} - 11.95~{\text{C}} + 0.25~{\text{AB}}~ + 17.59~{\text{AC}} + 1.58~{\text{BC}} - 17.61~{\text{A}}^{2} ~ - 0.41~{\text{B}}^{2} - 7.55~{\text{C}}^{2} - 1.58~{\text{ABC}}~~ - ~5.62~{\text{A}}^{2} {\text{C}}~ - ~9.99~{\text{AB}}^{2} ~ - ~0.27~{\text{B}}^{3} ~ + ~5.98~{\text{A}}^{2} {\text{B}}^{2}$$

**Table 2:** Experimental design matrix and the response of experimental settings for the reduction of Cr(VI) using ascorbic acid.

Run	Factors			Response	
	A:	B:	C:		
	Ascorbic acid (mg/L)	Humic acid (mg/L)	Cr(VI) (mg/L)	Cr(VI) residual (mg/L)	Cr(VI) reduction (%)
1	250.00	50.00	50.05	0.05	99.90
2	250.00	50.00	50.05	0.04	99.91
3	101.35	20.27	20.35	0.14	99.32
4	398.65	79.73	79.75	0.07	99.91
5	250.00	50.00	50.05	0.05	99.90
6	250.00	50.00	100.00	41.67	58.33
7	250.00	100.00	50.05	1.33	97.34
8	250.00	50.00	50.05	0.05	99.91
9	101.35	79.73	20.35	1.64	91.96
10	398.65	20.27	79.75	0.05	99.93
11	398.65	79.73	20.35	0.03	99.86
12	500.00	50.00	50.05	0.04	99.92
13	250.00	0.00	50.05	0.04	99.93
14	101.35	20.27	79.75	61.66	22.68
15	250.00	50.00	50.05	0.40	99.19
16	250.00	50.00	50.05	0.05	99.91
17	250.00	50.00	0.10	0.00	98.53
18	398.65	20.27	20.35	0.02	99.91
19	0.00	50.00	50.05	50.05	0.00
20	101.35	79.73	79.75	57.45	27.96

Equation (2) can help estimate the Cr(VI) reduction (%) as a function of ascorbic acid (A), humic acid (B), and initial Cr(VI) (C) concentration (mg/L). In the equation, the signs (positive/negative) of the regression coefficients represent the synergistic/antagonistic effect of the parameters on the response. The magnitudes of the coefficients are indicative of their relative impact on the response. It can be inferred from the equation that ascorbic acid (A) imparts the single biggest positive impact on the reduction of Cr(VI) with a coefficient of 29.71 in Eq. (2). While the initial Cr(VI) concentration (C) showed a negative impact on the Cr(VI) reduction (%). However, comparing the magnitude of the two coefficients, it was deduced that the relative impact of initial Cr(VI) concentration is less pronounced than the ascorbic acid dosage^31,36^.

The adequacy of the RSM model was statistically verified by performing the analysis of variance (ANOVA). The results of the ANOVA are summarized in Table 3. The statistical significance of the model and its terms were assessed by F-values and p-values. The high F-value along with the p-value below 0.0001 indicates the high statistical significance of the quartic model (i.e., over 99.99% confidence). As shown in Table 3, the p-values of all the terms were below 0.05 indicating their significance above 95% confidence level. The non-significant lack of fit justifies the adequate fitting of the model to the experimental data, thereby guaranteeing further confidence in the developed model^32,37^. The signal to noise ratio can be estimated from adequate precision value which compares the range of predicted values at the design points to the average prediction error^27,31^. Adequate precision value of above four is desirable to discriminate signal from noise. The model’s adequate precision value was more than two orders of magnitude higher than the desired value, thereby indicating the overwhelming abundance of signal when compared to noise for the development of the model. Consequently, statistical analyses establish the robustness of the RSM model.

**Table 3:** The analysis of variance (ANOVA) of the reduced quartic model representing the reduction of Cr(VI) using ascorbic acid.

Source of variation	Sum of squares	D_f_	Mean square	F-value		p-value	Significance
Model	18,307	13	1408	20,064		< 0.0001	Significant
A-Ascorbic acid	4992	1	4992	71,121		< 0.0001	
C-Cr(VI)	808	1	808	11,513		< 0.0001	
AB	1	1	1	7		0.037	
AC	2475	1	2475	35,266		< 0.0001	
BC	20	1	20	285		< 0.0001	
A^2^	3724	1	3724	53,063		< 0.0001	
B^2^	2	1	2	28		0.002	
C^2^	684	1	684	9745		< 0.0001	
ABC	20	1	20	283		< 0.0001	
A^2^C	105	1	105	1491		< 0.0001	
AB^2^	331	1	331	4716		< 0.0001	
B^3^	4	1	4	56		0.000	
A^2^B^2^	114	1	114	1626.900		< 0.0001	
Residual	0.421	6.000	0.070				
Lack of Fit	0.000	1.000	0.000	0.001		0.981	not significant
R^2^	1.000			Adjusted R^2^		0.999	
Coefficient of Variation (%)	0.312			Adeq. Precision		450.9	

**Figure 2:**
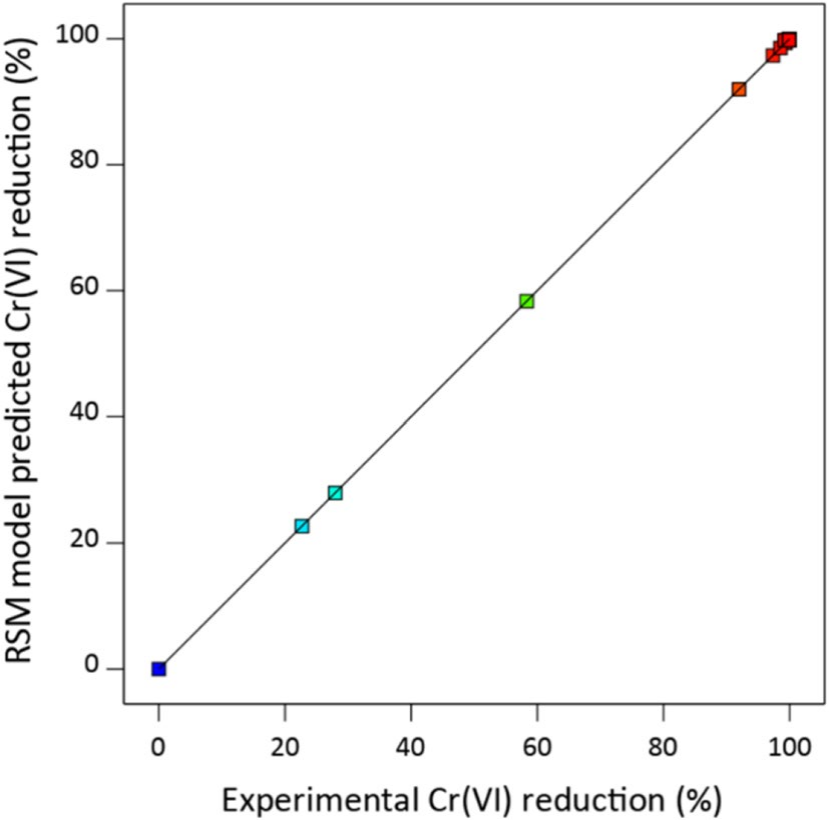
RSM model predicted versus experimental Cr(VI) reduction in water using ascorbic acid.

**Figure 3:**
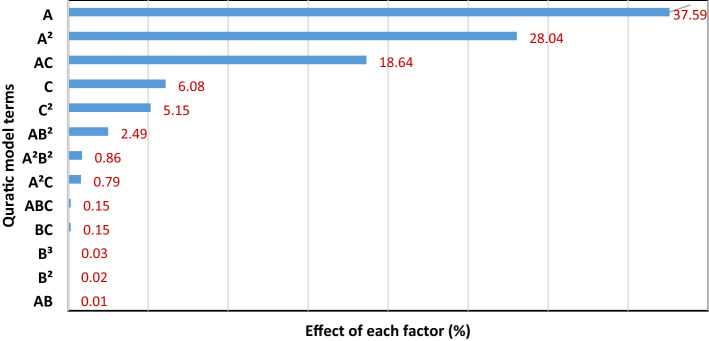
Graph of Pareto analysis representing the contribution of model terms on Cr(VI) reduction (%) response.

The predictability of the RSM model was assessed by plotting model predicted values against experimental Cr(VI) reduction (%), as shown in Fig. 2. The dataset yielded a perfect determination coefficient (R^2^ = 1) and a very high adjusted determination coefficient (adj. R^2^ = 0.999). This illustrates the high precision of the RSM model to describe the Cr(VI) reduction with respect to ascorbic acid, humic acid, and initial Cr(VI) concentration^31,37^. The contribution of each term towards the final model was calculated by performing the Pareto analysis illustrated in Fig. 3. The analysis establishes the highest contribution of ascorbic acid on Cr(VI) reduction and initial Cr(VI) concentration is ranked second. Humic acid was found to minimally influence the Cr(VI) reduction^38^.

### Effect of ascorbic acid, humic acid, and Cr(VI) initial concentration

**Figure 4:**
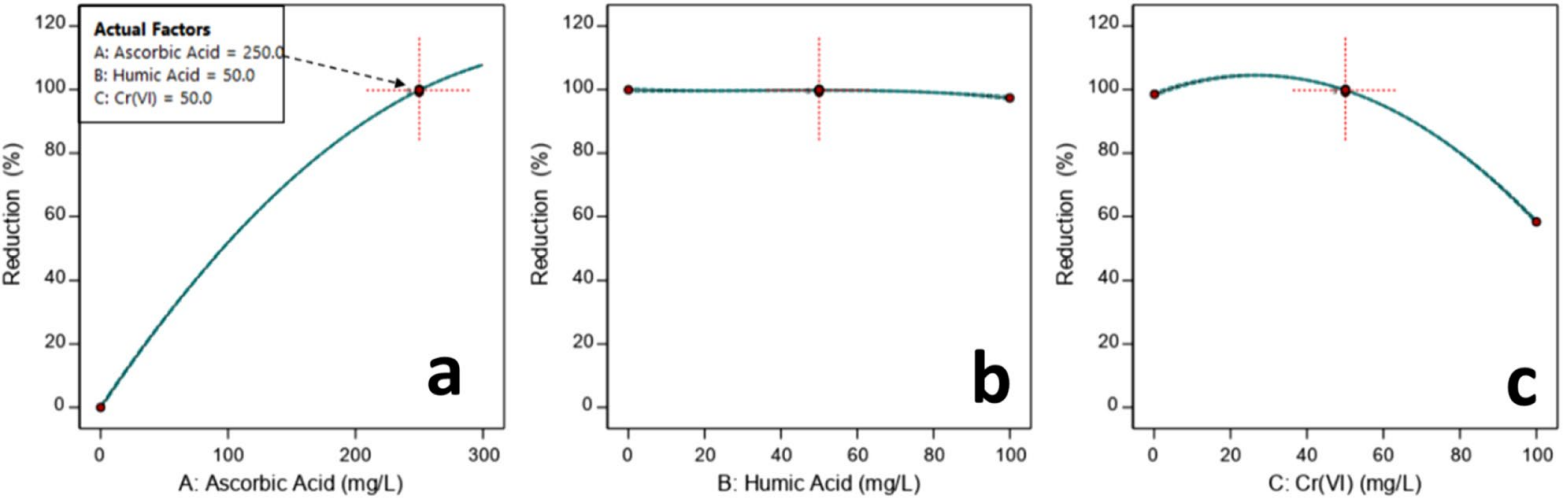
Effect of ascorbic acid, humic acid, and initial chromium concentration on the reduction of Cr(VI) in aqueous system.

The effects of the three parameters on Cr(VI) reduction were studied with the RSM plotting one-factor plots and three-dimensional (3D) surface plots. Figure 4 shows the one-factor plots to observe the individual effects of ascorbic acid (A), humic acid (B), and initial Cr(VI) (C) concentration on the reduction of Cr(VI) in the studied systems. The one-factor plots demonstrate the linear effect of varying the concentration of an individual factor while keeping the other two constant^28,32^. Figure 4a shows the rapid increase in Cr(VI) reduction with the increase in ascorbic acid dosage (0–300 mg/L). Nearly complete (≥ 99%) reduction of 50 mg/L initial Cr(VI) concentration was observed when the ascorbic acid dosage reached 250 mg/L in the presence of 50 mg/L humic acid as specified by the experimental design point in Fig. 4a. This experimental design point lies at the central level (0) of the experimental design space (Table 1) and corresponds to experimental runs 1, 2, 5, 8, 15 and 16 of the experimental design matrix (Table 2). This observation is in agreement with previously reported results^17^. The change in humic acid concentration (0–100 mg/L), however, does not significantly affect the Cr(VI) reduction in the studied systems as shown in Fig. 4b. Researchers have observed the reduction of Cr(VI) with humic acid alone^2,39,40^. They reported that Cr(VI) reduction by humic acid requires several weeks unlike the present study showing a rapid chemical reduction in 2 h^2^. The least impact of humic acid on Cr(VI) reduction supports our analysis of model terms. In Fig. 3, the term “B” (humic acid concentration) alone does not affect the model representing Cr(VI) reduction in the aqueous system. However, the terms representing interaction (e.g., AB and A^2^B^2^) contribute to a limited extent as shown in Eq. (2) and Fig. 3. Some literature also indicates the affirmative effect of reducing agents, such as ferric iron^39^ and zero-valent iron^40^, on the reduction kinetics of Cr(VI) in the presence of humic substances. Figure 4c shows the effect of Cr(VI) initial concentration on the reduction of Cr(VI) in the aqueous system. The graph shows a nearly complete (≥ 99%) reduction of Cr(VI) when the initial concentration of Cr(VI) is in the range of 0–50 mg/L. However, upon increasing the initial Cr(VI) concentration to within 50–100 mg/L the reduction ratio gradually decreases to 60%. This might be due to the scarcity of ascorbic acid for reducing higher concentrations of Cr(VI) beyond 50 (mg/L). Stoichiometrically, 1.42 mM ascorbic acid (250 mg/L) was able to reduce up to 0.961 mM Cr(VI) (50 mg/L) which is comparable to reported literature^17^. The effects of interaction between factors on Cr(VI) reduction are shown in Fig. S4 (Supplementary Information). The changes in humic acid concentrations (low: 20 mg/L, high: 80 mg/L) do not appear to affect the Cr(VI) reduction capacity of ascorbic acid (Fig. S4a) whereas those in initial concentrations of Cr(VI) significantly affect the reduction ratio (%) (Fig. S4b,c). Hence, one-factor plots postulate (i) the strong positive impact of ascorbic acid, (ii) practically no impact of humic acid, and (iii) the negative impact of initial concentrations of Cr(VI) on Cr(VI) reduction (%) in the aqueous system.

**Figure 5:**
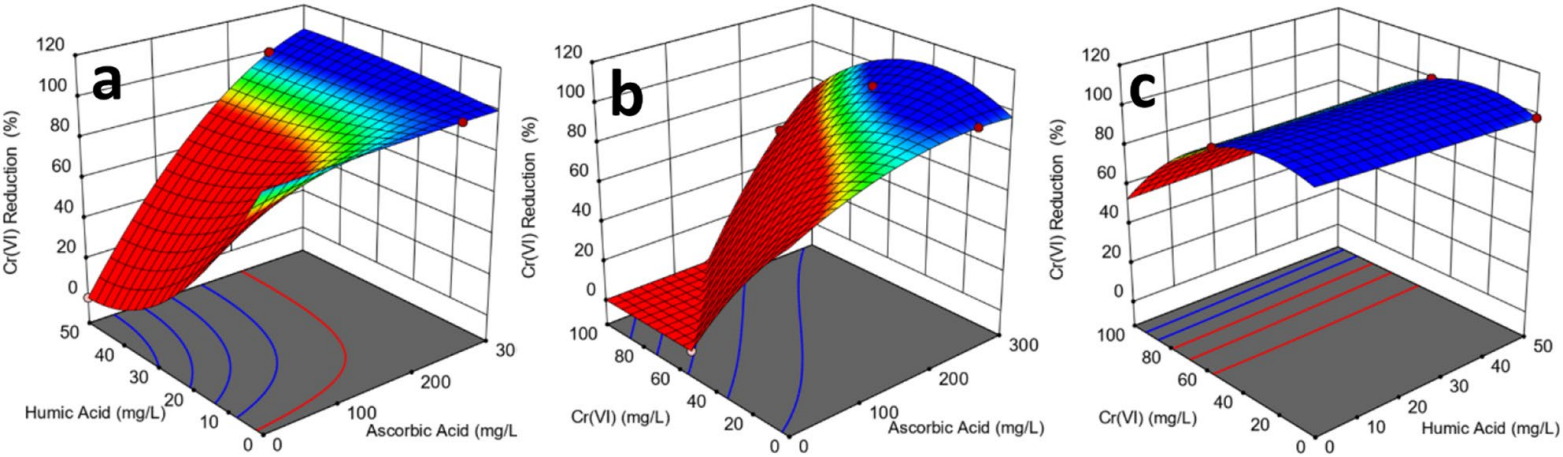
Response surface plots representing the interactive effects of (**a**) ascorbic acid and humic acid, (**b**) ascorbic acid and initial chromium concentration, and (**c**) humic acid and initial chromium concentration on the reduction of Cr(VI) in an aqueous system.

Figure 5 shows the interactive effects of two factors at a time on the Cr(VI) reduction in the aqueous system. The results of ANOVA (Table 3) indicate that the interaction terms (AB, AC, and BC) are statistically significant. Therefore, three-dimensional plots of Cr(VI) reduction against three possible combinations: (i) ascorbic acid and humic acid (Fig. 5a), (ii) ascorbic acid and initial Cr(VI) concentration (Fig. 5b), and (iii) humic acid and initial Cr(VI) concentration (Fig. 5c), were generated. Figure 5a–c shows various bands of response surfaces representing the extent of Cr(VI) reduction with the variance in ascorbic acid, humic acid, and initial Cr(VI) concentration. The 3D response surfaces might help estimate the combined effects of factors on Cr(VI) reduction, however, their utilization should be considered carefully. The standard error of design plots (Fig. S5 in Supplementary Information) warn of the increase in standard error beyond 0.45 at certain concentrations (ascorbic acid: (≤ 200–300 ≤) mg/L, humic acid (≤ 40–60 ≤) mg/L, and Cr(VI) (≤ 40–60 ≤) mg/L)^31^. Therefore, response surface plots will be more reliable within these concentration ranges^27,28,31^. Nevertheless, they can be helpful in visualizing the RSM model developed to represent the Cr(VI) reduction in aqueous systems^10,38^.

### Optimization of Cr(VI) reduction and reduction kinetics in water

The ultimate objective of this study was to identify the optimum ascorbic acid dosage required to completely reduce Cr(VI) in the presence of environmental concentrations of humic acid (i.e. 20 mg/L)^41^. From an application standpoint, process optimization is vital to achieve the highest efficiency of contaminant removal from water. Therefore, operational parameters such as Cr(VI) initial concentration, adsorbent dose, and concentration of competing substances (i.e. humic acid in wastewater) were optimized. The role of pH on the reduction of Cr(VI) is well established in literature^40,42,43^, therefore, this study was not aimed at investigating the effect of pH during ascorbic acid reduction. Instead, this work explored the optimal reduction of Cr(VI) from actual water and wastewater systems where the pH is naturally buffered and the presence of humic acid is difficult to avoid. Therefore, the pH of the system was not altered despite its significant impact on Cr(VI) reduction. Whereas, though insignificant, the impact of humic acid was included throughout the study. Four initial concentrations of Cr(VI) were selected to address the entire range of Cr(VI) in surface water, contaminated groundwater, and sewage and industrial wastewater as shown in Table 4. Numerical optimization was performed by employing an objective (desirability) function to predict the ascorbic acid dosages to completely reduce Cr(VI) in aqueous systems. The RSM model (Eq. 2) was used to predict the optimum ascorbic acid dosages. Verification experiments were performed to observe the Cr(VI) reduction at the optimized dosages. The difference between model predicted and experimentally observed Cr(VI) reduction in DIW was < 0.4% for all cases as shown in Table 4. This indicates the suitability of the model for usage in the Cr(VI) reduction by ascorbic acid in aqueous systems in the presence of humic acid.

**Table 4:** Optimized reduction of Cr(VI) in water.

Cr (VI) conc. (mg/L) [Influent]	Optimum ascorbic acid (mg/L)	Humic acid (mg/L)	Target reduction (%)	Experimental Cr(VI) reduction (%)	Error (%)
1	267	20	100	99.65	0.346
10	323	20	100	99.89	0.111
20	394	20	100	99.91	0.090
100	500	20	100	99.93	0.062

**Figure 6:**
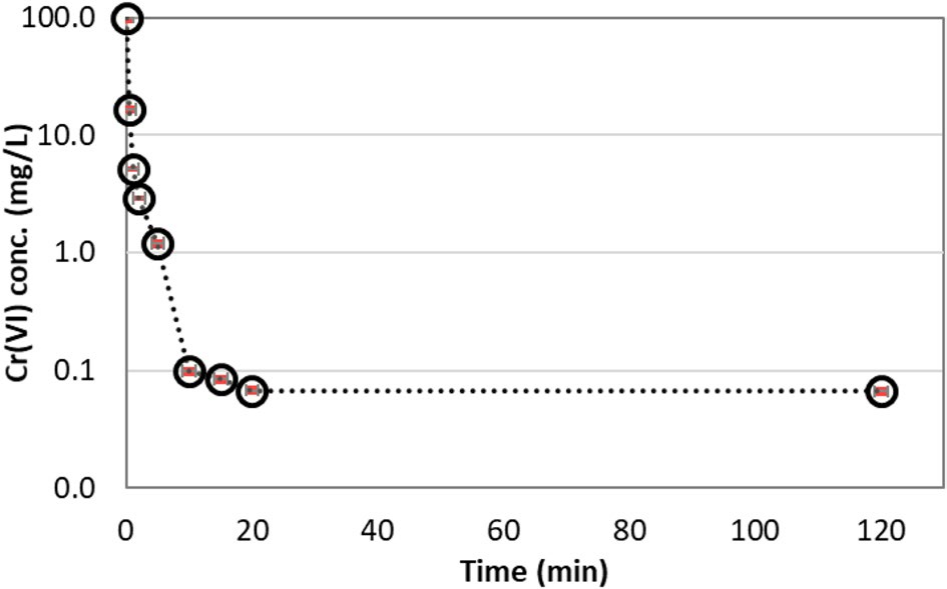
Reduction kinetics of Cr(VI) (100 mg/L) by ascorbic acid (500 mg/L) in the presence of humic acid (20 mg/L).

The rate of Cr(VI) reduction by ascorbic acid was determined by observing reduction kinetics at optimized parameters. The experiments were performed by adding 500 mg/L ascorbic acid to 100 mg/L Cr(VI) aqueous solution containing 20 mg/L humic acid (Table 4). The 20 mg/L humic acid was selected due to the prevalence of this typical concentration of humic acid in natural surface waters^41^. Initially, the reduction rate of Cr(VI) was very rapid and the concentration of Cr(VI) decreased from 100 to 1 mg/L in the first 5 min, as shown in Fig. 6. This rapid reduction rate continued until the residual Cr(VI) concentration approached 0.07 mg/L in the contact time of 20 min. The system was periodically monitored for 2 h and further Cr(VI) reduction was not observed. Stoichiometrically, 1.92 mM Cr(VI) was reduced by 2.8 mM of ascorbic acid. These values are very close to those calculated by Xu et al.^17^. They observed that, irrespective of acidic or alkaline aqueous environment, three moles of ascorbic acid were utilized to reduce one mole of dichromate, i.e., two moles of Cr(VI). The reduction kinetics followed pseudo second order rate reaction and the rate constant was 0.72 mM^-1^.sec^-1^. Our observations corresponded well with previous studies reducing Cr(VI) with ascorbic acid in DIW^17,26^.

### Cr(VI) reduction in various waters and environmental implications

**Figure 7:**
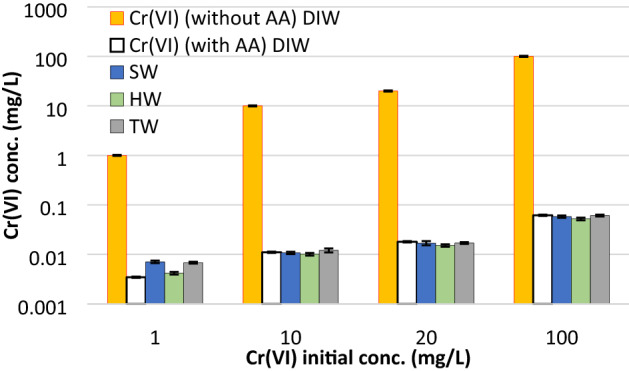
Reduction of Cr(VI) in the presence of optimized concentrations of ascorbic acid in various waters (*DIW* deionized water, *SW* synthetic soft water, *HW* synthetic hard water, *TW* real tap water). The reduction was over 99.5% in all waters and the residual concentration of Cr(VI) was below 0.1 mg/L in all cases.

The optimized dosages of ascorbic acid (Table 4) were utilized to observe the reduction capacity of ascorbic acid in SW, HW, and TW. The description of these waters can be found in the materials and methods section and their detailed ionic composition can be determined from Smith et al.^29^. Figure 7 shows the residual concentrations of Cr(VI) in various waters after the addition of the optimum dosage of ascorbic acid predicted from the RSM model (Eq. 2) and shown in Table 4. It was observed that over 99.5% Cr(VI) was reduced by ascorbic acid in all types of water. The residual Cr(VI) concentration was below 0.1 mg/L in all four studied aqueous systems.

This study systematically explores the utilization of ascorbic acid for the reduction of Cr(VI) in various waters. Ascorbic acid is a biologically safe and economically inexpensive chemical compound (~ $ 1/kg)^44^. Though the regulatory authorities (such as EPA and WHO) encourage the removal of both Cr(III) and Cr(VI), it is an established scientific fact that Cr(III) is less soluble and far less toxic than Cr(VI)^6^. Therefore, once Cr(VI) is reduced to Cr(III), it can be easily removed from water by hydroxide precipitation^45^. The precipitated chromium can be reused in chromium plating, leather tanning, and/or other industries.

## Conclusions

Over 99.5% reduction of the initial Cr(VI) concentration was achieved through the addition of an optimal amount of ascorbic acid in various waters. RSM was successfully applied to systematically study the effects of ascorbic acid, humic acid, and initial Cr(VI) concentration on Cr(VI) reduction in aqueous systems. An empirical model was developed, statistically verified, and experimentally validated to establish the relationship between Cr(VI) reduction in water and the factors affecting it. The individual and combined effects of factors affecting Cr(VI) reduction in the aqueous system were graphically demonstrated. It was observed that the Cr(VI) reduction in water chiefly depends upon ascorbic acid dosage followed by the Cr(VI) initial concentration. The humic acid concentration negligibly impacts Cr(VI) reduction in water over a short time (≤ 2 h). The 1, 10, 20, and 100 mg/L Cr(VI) solutions can be reduced to < 0.1 mg/L in DIW, SW, HW, and TW by adding 267, 323, 394, and 500 mg/L ascorbic acid, respectively. After ascorbic acid mediated reduction of Cr(VI), the less soluble Cr(III) could be recovered from the aqueous system via precipitation using hydroxide. These results could serve as a basis for improving sustainable Cr(VI) reduction from industrial wastewaters and might be adopted for Cr(VI) recovery and reuse. This study can be helpful in (i) treating low concentrations of Cr(VI) in drinking water and/or (ii) developing an environmentally benign system to economically reduce Cr(VI) to Cr(III) for precipitation and reuse in industrial wastewaters.

## Supplementary Information

Supplementary Information.
