# Supplementary material for: Experimental modeling and optimization for the reduction of hexavalent chromium in aqueous solutions using ascorbic acid
Source: Sci Rep. 2021 Jun 23;11:13146. doi: 10.1038/s41598-021-92535-y (PMC8222380; doi:10.1038/s41598-021-92535-y)
Supplement: Supplementary file 1 — Supplementary Information. [file 41598_2021_92535_MOESM1_ESM.docx]

**Supplementary Information**

Experimental modeling and optimization for the reduction of hexavalent chromium in aqueous solutions using ascorbic acid

Qammer Zaib ^1^, Hung Suck Park ^1^, Daeseung Kyung ^1,*^

^1^ Department of Civil and Environmental Engineering, University of Ulsan, Daehak-ro 93, Nam-gu, Ulsan 44610, Republic of Korea.

*Corresponding author:

Tel: +82-52-259-2259, Fax: +82-52-221-0152

E-mail: dkyung@ulsan.ac.kr

**Quantitative verification of Cr(VI) and Cr(III) in aqueous solutions**

To ensure the mass balance of chromium in the aqueous solutions (deionized water, soft water, hard water, and tap water), quantitative ratios of Cr(VI) and Cr(III) were determined. ICP-OES quantified total chromium in a sample and EPA Method 7196 was opted to determine Cr(VI) concentrations ^1,2^. The difference of the two concentrations yielded the Cr(III) concentration^2^. Figure S1 and S2 represent the standard calibration curves obtained by running standard solutions of total chromium and Cr(VI), respectively. These curves were used to quantify the concentration of total chromium and Cr(VI) in working solutions. The total chromium standard curve was obtained by running five standards (0-10 mg/L), whereas the Cr(VI) standard curve was obtained by running thirteen standards (0-100) mg/L. Both standard curves exhibited regression coefficients of ≥ 0.99, justifying their suitability for usage. The speciation of Cr(VI) and Cr(III) in aqueous solutions is shown in Fig. S3. The dominant species is Cr(VI) before the addition of ascorbic acid. The optimum dosage of ascorbic acid (350 mg/L), determined from numerical optimization, largely reduced Cr(VI) to Cr(III) in all water types. The residual Cr(VI) in aqueous solutions is below 0.01 mg/L after the addition of optimum dosage of ascorbic acid.

**Figure S1.** Calibration curve used to quantify the concentration of total chromium in aqueous solutions.

**Figure S2.** Calibration curve used to quantify the concentration of Cr(VI) in aqueous solutions.

**Figure S3.** Chromium speciation with and without adding 350 mg/L (optimum dosage) of ascorbic acid in studied water solutions: (DIW = deionized water, SW = soft water, HW = hard water, TW = real tap water). The red bar for DIW (w/o AA) indicates total chromium as a form of Cr(VI) before the addition of ascorbic acid.


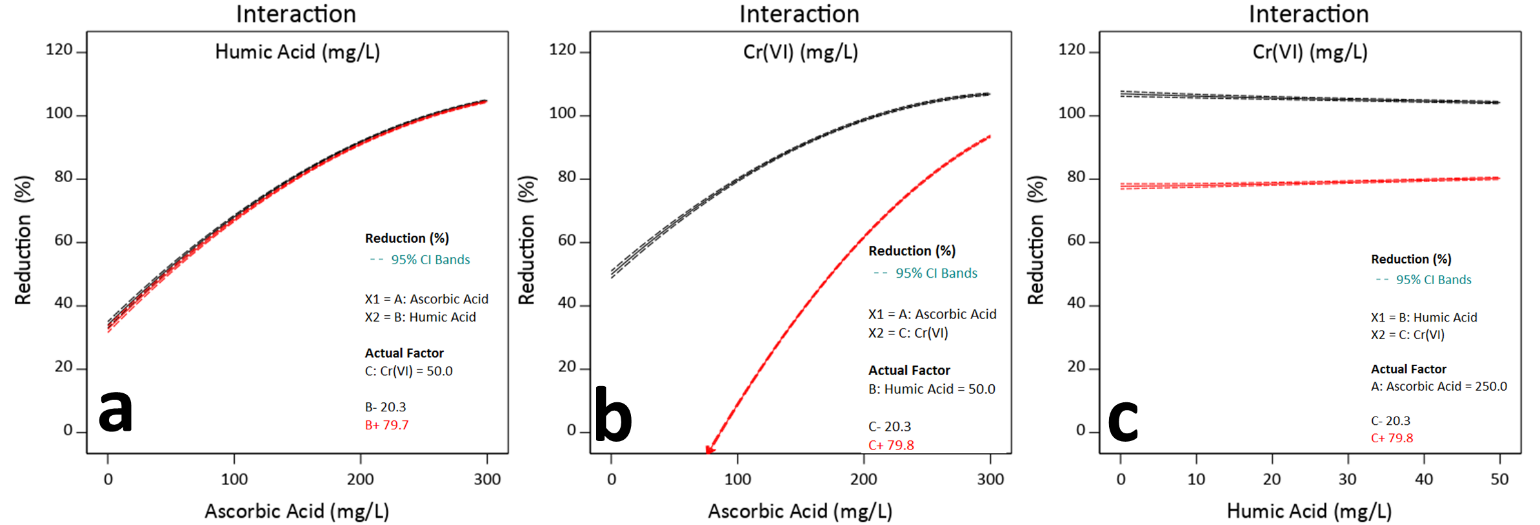


**Figure S4.** Interactions of experimental factors and their effect on Cr(VI) reduction


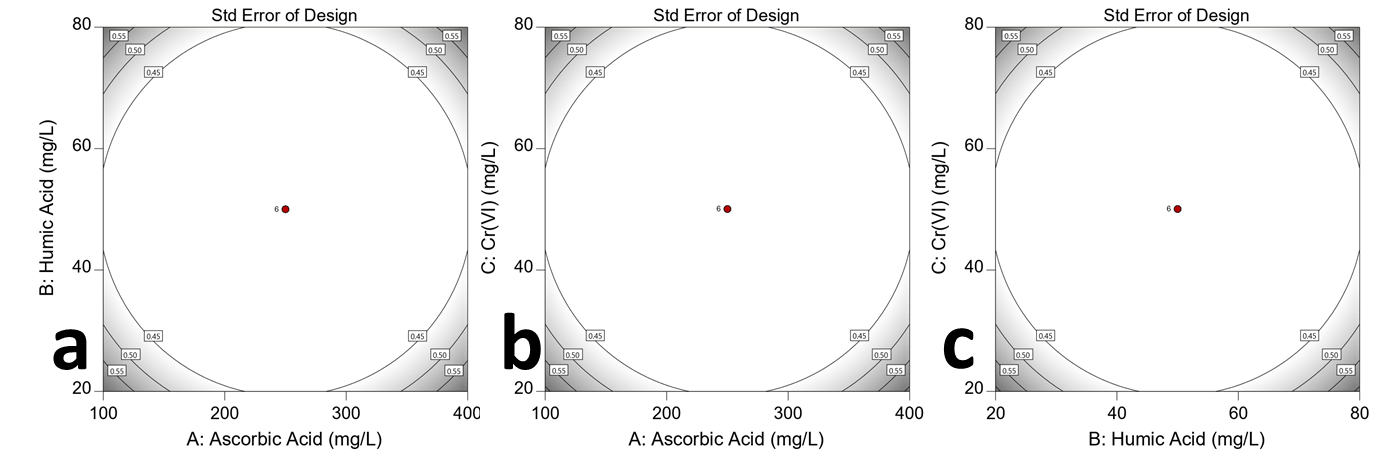


**Figure S5.** The standard error of design for the model representing the combined effect of experimental factors on Cr(VI) reduction.

**References**

1. United States Environmental Protection Agency Method 7196A Chromium Hexavalent (Colorimetric). 1–6 (1992).

2. Zewdu, F. & Amare, M. Determination of the level of hexavalent, trivalent, and total chromium in the discharged effluent of Bahir Dar tannery using ICP-OES and UV–Visible spectrometry. *Cogent Chem.* **4**, 1534566 (2018).
